# Gallstone Ileus Secondary to Cholecysto‐Duodenal Fistula in a Middle‐Aged Female: A Rare Surgical Emergency and Review of the Literature

**DOI:** 10.1002/ccr3.73027

**Published:** 2026-06-25

**Authors:** Hervé Tshikomba Mbuyamba, Charles Oswald Ruboha, Thomas Ruai Chol, Bahdon Mohamed Ali, Carlos Kasole Balole, Maria Shemweta, Michael Jimmy Igoti, Michael Machage, Balowa Musa, Daniel William Kitua, Godfrey Masalu Mchele, Ally Hamis Mwanga, Séverin Akinja Uwonda

**Affiliations:** ^1^ Department of Surgery, School of Medicine Muhimbili University of Health and Allied Sciences Dar es Salaam Tanzania; ^2^ Department of Surgery, School of Medicine Université Officielle de Mbujimayi Mbujimayi Democratic Republic of the Congo; ^3^ Department of Radiology, School of Medicine Muhimbili University of Health and Allied Sciences Dar es Salaam Tanzania; ^4^ Department of Surgery Muhimbili National Hospital Dar es Salaam Tanzania

**Keywords:** cholecystectomy, cholecystoenteric fistula, cholelithiasis, duodenal repair, enterotomy, gallstone ileus

## Abstract

Gallstone ileus secondary to cholecysto‐duodenal fistula is a serious and uncommon complication due to cholelithiasis, characterized by mechanical bowel obstruction due to gallstone impaction following a spontaneous biliary‐enteric fistula. The case highlights the diagnostic challenge and the importance of clinical evaluation and thorough investigation of the patients.

## 1 Introduction

Gallstone ileus is an intestinal obstruction secondary to the presence of one or many gallstones in the lumen of intestines [1, 2, 3]. It is a rare condition accounting for approximately 1%–4% of all cases of bowel obstruction [3]. Gallstone ileus (GI) is a rare gallstone complication estimated to occur in up to 25% among patients over 65 years old with a history of gallstone disease [3]. It arises from the formation of a biliary‐enteric fistula, typically cholecysto‐duodenal, allowing large gallstones to enter the bowel and cause obstruction, most frequently in the terminal ileum [4, 5, 6]. The mortality associated with GI ranges between 12% and 27% [1, 7]. Diagnosis is often delayed due to nonspecific symptoms, particularly in patients without known biliary pathology [2]. Classical findings on plain abdominal radiography include: pneumobilia; intestinal obstruction; an aberrantly located gallstone; and [4] change of location of a previously observed stone. The optimal management of acute GI is controversial and is basically surgical [1].

## 2 Case History and Examination

A 47‐year‐old female presented with colicky abdominal pain for 7 days, inability to pass stool for 4 days, four episodes of non‐projectile bilious vomiting, and progressive abdominal distension. There was neither comorbidity nor prior history of gallstone disease. She was a referral from a peripheral hospital where an abdominal CT‐Scan was done.

On examination, she was alert, afebrile, not jaundiced, not pale, not dyspneic. The body mass index was calculated to 34 kg/m^2^. Vital signs indicated tachycardia (heart rate of 126 beats/min), with other parameters within the normal ranges. The abdomen was distended generally with sluggish bowel sounds, and no palpable abdominal mass. Digital rectal examination revealed an empty rectum. Other systems were essentially normal.

## 3 Differential Diagnosis, Investigations, and Treatment

The laboratory workout showed leukocytosis (WBC:13,780 K/μL) with shift to left and mild anemia (Haemoglobine:11.54 g/dL, Hematocrit: 38.54%, Mean cell volume: 79.96 fL, Mean cell hemoglobin: 23.94 pg., Mean cell hemoglobin concentration: 29.94 g//dL). Liver function tests, and electrolyte levels were normal.

The CT scan images were reviewed at our facility. They revealed the classic Rigler's triad (**Figure** **1** **A–F**). A diagnosis of intestinal obstruction, with suspicion of gallstone ileus was made. Initial resuscitation was done by insertion of intravenous cannulas and administration of intravenous fluids, insertion of urinary catheter and nasogastric tube, intravenous antibiotics, and the patient was kept nil per mouth while preparing her for exploratory laparotomy.

**FIGURE 1:**
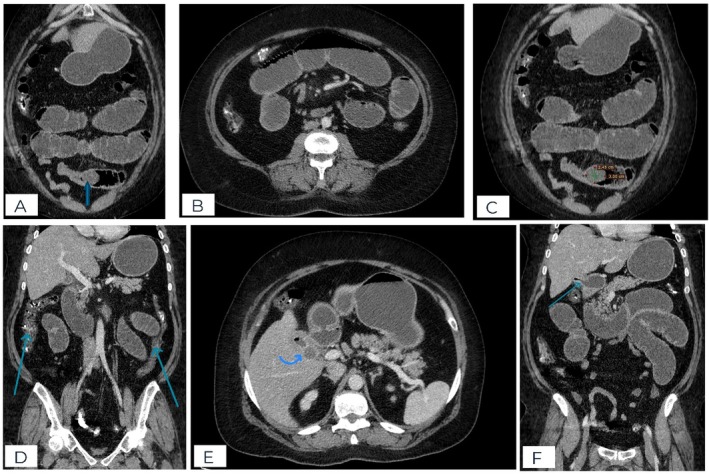
(A–F) Contrast‐enhanced abdominal CT scan and contrast enema (axial and coronal planes) demonstrate features consistent with small bowel obstruction, with a transition point near to the terminal ileum (thick blue arrow). At this level, a round, subtle laminated structure measuring 3.00 × 2.45 cm, consistent with a gallstone, is identified. There is marked dilatation of the proximal small bowel loops with multiple air–fluid levels and associated gastric distension. Distal to the transition point, the remaining small bowel and large bowel loops (thin long arrows) are collapsed. The gallbladder appears shrunken and contains a small amount of intraluminal air, consistent with pneumobilia (curved arrow). A relatively subtle cholecystoenteric fistula (thin short arrow) is demonstrated between the shrunken gallbladder and the duodenum (Images A–F).

An emergency open laparotomy was subsequently done using an extended midline incision. The findings were: Serous yellowish peritoneal fluids, grossly dilated small bowel (Figure 2), impacted gallstone measuring about 3 × 2.5 cm located at 62 cm from the iliocaecal junction (Figure 3), cholecysto‐duodenal fistula (Figure 4), and contracted thickened gallbladder with adhesions. Enterotomy was performed in a non‐inflammatory healthy area, through which the stone was extracted (Figure 5A,B). The opening was then closed in double layers using vicryl 3/0. Cholecystecmoy was done, followed by the refreshment and primary repair of duodenal fistula. The duodenal defect was closed using the modified Graham's omental patch technique. Peritoneal lavage was performed, followed by tubular drain placement and multi‐layered abdominal closure. Postoperatively the patient was kept nil per oral for 48 h; IV fluids and antibiotics were given, and the abdominal drain was removed on postoperative Day 4. The patient was discharged on Day 5. No postoperative complication was encountered as the follow‐up was uneventful during one year as an outpatient.

**FIGURE 2:**
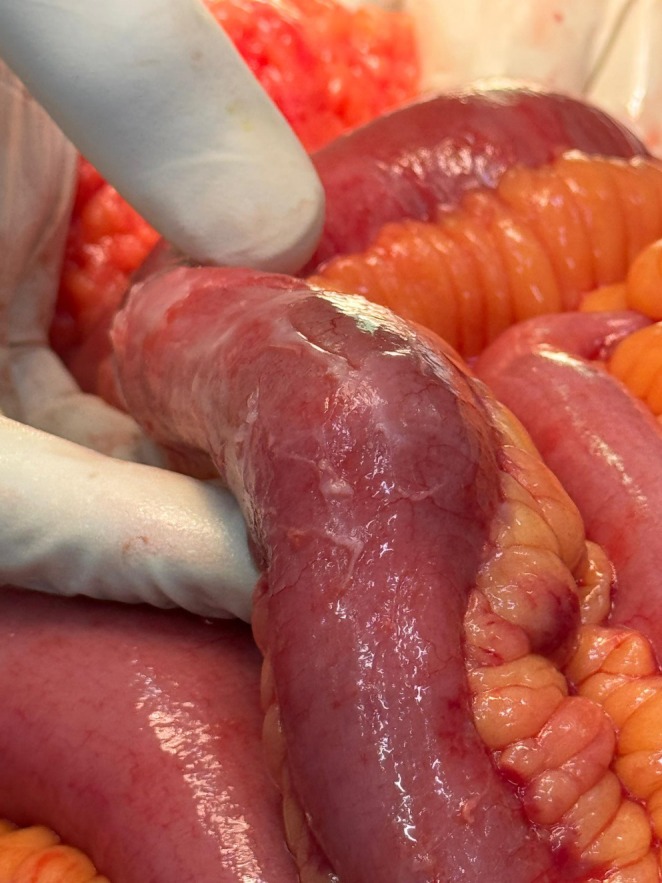
Grossly distended small bowels.

**FIGURE 3:**
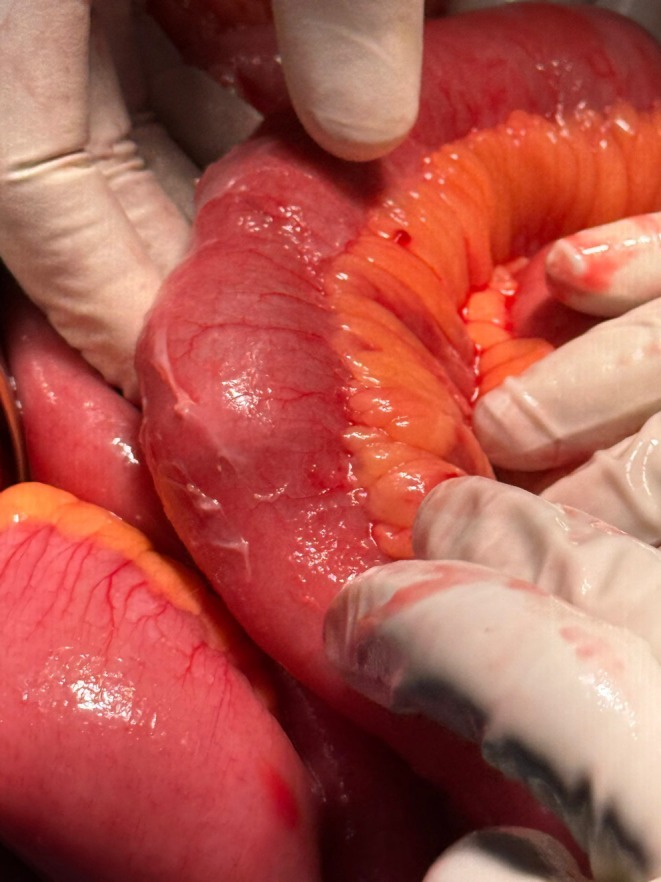
Area of impacted gallstone.

**FIGURE 4:**
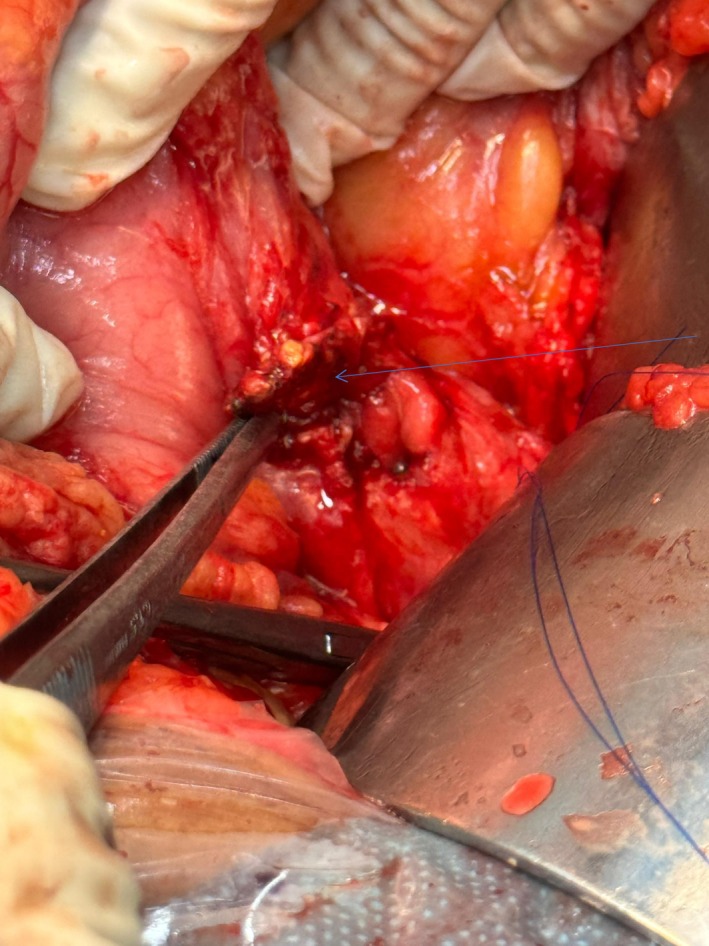
Duodenal fistula (Blue arrow).

**FIGURE 5:**
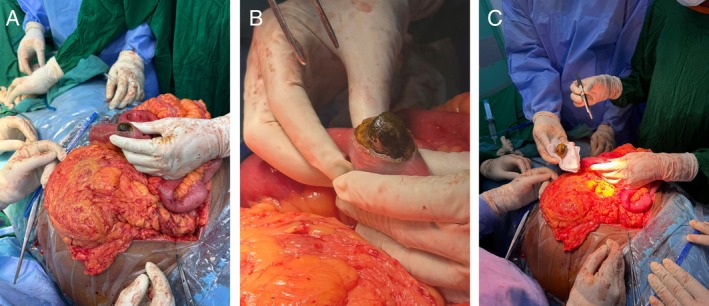
(A) Enterotomy showing the impacted gallstone. (B) Extraction of the impacted gallstone after enterotomy. (C) Extracted gallstone that was causing bowel obstruction.

## 4 Conclusion and Results

Although a rare case, there is a need to consider gallstone ileus in cases of bowel obstruction, even among middle‐aged patients without prior biliary complaints. Thorough investigations and timely surgical intervention can result in excellent outcomes when diagnosis is suspected early.

## 5 Discussion

Gallstone ileus presented a challenging surgical dilemma [8]. While typically presenting in elderly females with a known history of cholelithiasis, this case involved a younger patient without prior biliary symptoms. Diagnosis is frequently delayed due to vague symptoms and low clinical suspicion [6]. In this case, however, the patient was a multiparous female above 40 years, with a body mass index of 34 kg/m^2^. These were important risk factors that should be considered even though she did not report any prior history of gallstone disease. The case was misdiagnosed at a peripheral hospital despite abdominal CT‐scan that was done. Misdiagnosis is common and it also carries a significant rate of complications with a mortality rate ranging from 12% to 27% in most series reported [9].

Gallstone ileus is a mechanical obstruction of the gastrointestinal tract [1, 2, 3]. The clinical manifestation will depend on the site of obstruction. Usually, the gallstone obstructs the ileocaecal junction, leading to Bernard's syndrome [7, 10]. Fewer than 4.8% of the patients present with colonic obstruction, whereas 3% of the patients can present with Bouveret's syndrome, which is the result of gallstone lodging in the duodenum and blocking the gastric outlet [11]. The gallstone may cause colonic obstruction in the case of cholecystocolonic fistula with migration of the gallstone into the colonic lumen. Some cases of sigmoid gallstone ileus have been reported [6]. In our case report, the gallstone was impacted at 62 cm from the ileocaecal junction (Figure 4).

Imaging such as abdominal CT or X‐ray may demonstrate Rigler's triad: pneumobilia (Gotta‐Montschler sign), ectopic gallstone, and intestinal obstruction [8]. Evidence of change in gallstone location in the second CT or X‐ray is known as Rigler's tetrad [7]. The presence of 2 of the 3 signs is diagnostic [7]. The features of Rigler's triad were previously missed out during interpretation of images from the contrast‐enhanced abdominal CT scan and contrast enema that was done at a peripheral hospital. These have been diagnosed at our facility when the images were reviewed by radiologists (Figure 1A–F). Traditionally, plain abdominal radiography remained a mainstay for the assessment of small bowel obstruction. However, the sensitivity of plain film varies from 40% to 70% in diagnosing gallstone ileus [12, 13]. The CT scan remains the most sensitive and specific imaging modality for diagnosing gallstone ileus, with reported accuracy up to 93% [7, 8].

Gallstone ileus with cholecystoenteric fistula is classified as type Vb of Mirizzi syndrome as per modified Csendes classification validated by Beltran and Csendes in 2008 [14]. The purpose of different classifications of a disease is to guide the management. However, Mirizzi syndrome type Vb is a matter of controversy. Some authors advise to treat the acute condition first (gallstone ileus) and at a second time after the patient has recovered from surgery (3 or more months later), approach the gallbladder according to the presence or absence of external compression of the bile duct or cholecystobiliary fistula [15, 16]. Practically the surgical management of the gallstone ileus can include: (1) enterotomy with stone extraction alone; (2) enterotomy, stone extraction, cholecystectomy and fistula closure; (3) bowel resection alone; and (4) bowel resection with fistula closure [1, 17, 18, 19]. The choice of surgical procedure is determined largely by clinical status. GI patients are usually elderly and have comorbidities, so enterotomy with stone extraction alone appears to be more suitable than more invasive techniques [1, 19, 20]. This case is about a 47‐year‐old female patient without comorbidities. The gallstone ileus was addressed with enterotomy followed by gallstone extraction after cholecystectomy was done. Duodenal defect was refreshed and primarily closed. The one‐stage surgery was justified by the age, intraoperative findings, and the clinical status of the patient. Otherwise, conservative management has been traditionally advised in gallstones measuring less than 2.5 cm with spontaneous resolution of gallstone ileus being reported for gallstone measuring 2.6 cm [5].

## Author Contributions

**Hervé Tshikomba Mbuyamba:** conceptualization, data curation, formal analysis, investigation, methodology, project administration, resources, software, validation, visualization, writing – original draft, writing – review and editing. **Charles Oswald Ruboha:** conceptualization, data curation, formal analysis, resources, visualization, writing – original draft, writing – review and editing. **Thomas Ruai Chol:** conceptualization, data curation, writing – original draft, writing – review and editing. **Bahdon Mohamed Ali:** data curation, formal analysis, methodology, visualization, writing – review and editing. **Carlos Kasole Balole:** data curation, formal analysis, investigation, resources, visualization, writing – review and editing. **Maria Shemweta:** data curation, formal analysis, investigation, methodology, software, validation, visualization, writing – review and editing. **Michael Jimmy Igoti:** data curation, resources, software, visualization, writing – review and editing. **Michael Machage:** data curation, formal analysis, resources, software, visualization, writing – review and editing. **Balowa Musa:** data curation, formal analysis, investigation, methodology, resources, software, validation, visualization, writing – review and editing. **Daniel William Kitua:** formal analysis, investigation, methodology, validation, visualization, writing – original draft, writing – review and editing. **Godfrey Masalu Mchele:** conceptualization, data curation, formal analysis, investigation, methodology, project administration, resources, validation, visualization, writing – review and editing. **Ally Hamis Mwanga:** formal analysis, methodology, supervision, validation, visualization, writing – review and editing. **Séverin Akinja Uwonda:** formal analysis, methodology, project administration, supervision, validation, visualization, writing – review and editing.

## Funding

The authors have nothing to report.

## Ethics Statement

This case report was therefore exempt from our institution's review board's ethical approval process as it reports a single rare clinical case.

## Consent

The written informed consent was obtained from the patient for this case report to be published in accordance with the journal regulations.

## Conflicts of Interest

The authors declare no conflicts of interest.

## Data Availability

The corresponding author can provide the data supporting the study's conclusions upon request. Due to ethical and privacy concerns, the data are not publicly accessible.
